# A technology for the investigation of biofilm transmission under shearing pressures

**DOI:** 10.1111/1751-7915.12848

**Published:** 2017-08-25

**Authors:** Miguel A. Matilla

**Affiliations:** ^1^ Department of Environmental Protection Estación Experimental del Zaidín Consejo Superior de Investigaciones Científicas Prof. Albareda 1 Granada 18008 Spain

## Abstract

Biofilm formation is a multifactorial and dynamic process. Stages of biofilm formation are highly regulated and include bacterial attachment to a target surface, formation of microcolonies, biofilm maturation and dispersion. This article highlights recent research by Gusnaniar *et al*., (2017) in which the authors develop a device to investigate bacterial biofilm transmission between surfaces under shearing pressures. The instrument can potentially be used to investigate the role of different genetic determinants and environmental cues on biofilm stability and transmission.

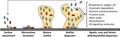

Originally, bacteria were referred to as planktonic microorganisms. However, they are commonly found associated with a broad range of biotic and abiotic surfaces forming complex and structured communities known as biofilms. One of the earliest descriptions of a biofilm was reported in the early 1940s (Heukelekian and Heller, 1940), corroborating earlier observations by Antonie van Leewenhoek in which he identified bacteria growing on tooth surfaces (Donlan, 2002; Lane, 2015). Bacteria in biofilms are generally embedded in a self‐produced extrapolymeric matrix that mainly consists of polysaccharides, proteins, lipids and nucleic acids. This extracellular matrix is responsible for providing adhesiveness, cohesiveness, stability and three‐dimensional architecture to the biofilm (Abee *et al*., 2011; Domenech *et al*., 2012; Flemming, 2016; Rice *et al*., 2016).

In nature, bacterial biofilms are involved in multiple biogeochemical processes and, as a result, they are commonly used in different biotechnological applications (Halan *et al*., 2012; Smith *et al*., 2015; Flemming *et al*., 2016). However, biofilms are also associated with numerous problems at industrial, clinical and agricultural levels, being responsible for major industrial contaminations and persistent infections in humans, animals and plants. In fact, current estimations indicate that around 80% of human infections are associated with the development of biofilms (Donlan, 2002; Romling and Balsalobre, 2012; Guilhen *et al*., 2017). This human health problem is often due to increased resistance of biofilms to different environmental stresses and antimicrobial agents (Davies, 2003; Van Acker *et al*., 2014; Flemming *et al*., 2016). Importantly, natural biofilms typically consist of multiple bacterial species, and some studies have demonstrated that mixed biofilms exhibit greater resistance to stressors than single‐species biofilms (van der Veen and Abee, 2011; Lee *et al*., 2016; Rice *et al*., 2016).

Biofilm formation is a complex phenomenon, that is generally divided into several stages. The process starts with the approach and attachment of bacterial cells to the target surface. This initial attachment is followed by cell multiplication, formation of microcolonies and the development of a mature biofilm. In late stages of biofilm development, cells detach from the biofilm allowing bacterial dissemination and the subsequent colonization of new niches (Fig. 1). As a consequence, this detachment favours bacterial survival and, in the case of bacterial pathogens, disease progression (O'Toole *et al*., 2000; Yildiz, 2007; Kaplan, 2010; Guilhen *et al*., 2017). Biofilm dispersal can be an active or passive process. The latter mainly refers to cell detachment caused by external physical forces such as fluid shearing, abrasion or mechanical interventions. Alternatively, active dispersal is triggered by the bacteria themselves and involves the sensing of environmental cues (i.e. changes in nutrient availability, pH, temperature and oxygen levels; nitric oxide, D‐amino acids) together with the recognition of intercellular and intracellular signals (i.e. acyl‐homoserine lactones, *cis*‐unsaturated fatty acids, autoinducers 2). The sensing of these signals may result in the modulation of downstream transduction pathways (Kaplan, 2010; Abee *et al*., 2011; Domenech *et al*., 2012; Petrova and Sauer, 2016; Guilhen *et al*., 2017). Thus, regulatory pathways involving quorum sensing (QS), bacterial second messengers (i.e. c‐di‐GMP, (p)ppGpp) and small regulatory RNAs have been shown to be involved in the modulation of biofilm dispersal though different mechanisms (Kaplan, 2010; Petrova and Sauer, 2016; Guilhen *et al*., 2017). Among these mechanisms, active biofilm detachment can be promoted by the synthesis of matrix‐degrading enzymes (i.e. hydrolases, proteases, deoxyribonucleases) and production of surfactants (i.e. rhamnolipids, viscosin) (Fig. 1) (Kaplan, 2010; Petrova and Sauer, 2016; Fleming and Rumbaugh, 2017; Guilhen *et al*., 2017).

**Figure 1:**
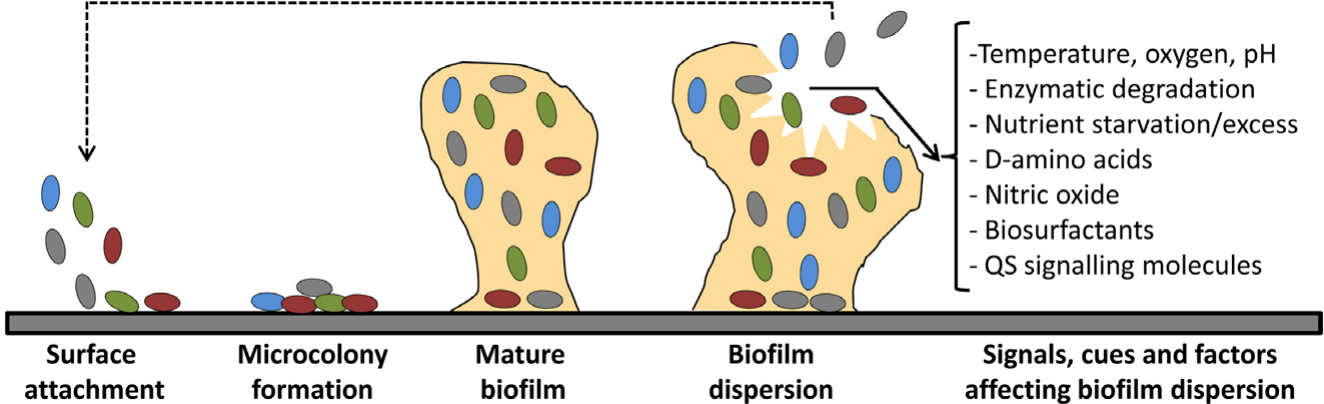
Stages of biofilm formation in bacteria and signals modulating biofilm dispersion.

Biofilm transmission generally requires both the detachment of bacterial cells from a mature biofilm and the attachment of dispersed cells into the new surface. Among the mechanical forces affecting biofilm transmission, shearing is one of most common passive mechanisms (Donlan, 2002; Hall‐Stoodley and Stoodley, 2005; Kaplan, 2010). In this issue of Microbial Biotechnology, Gusnaniar *et al*. (2017) describe the development of an instrument to investigate biofilm transmission from stainless donor surfaces to silicone rubber tubes under shearing conditions; a device that can be potentially adapted to the investigation of biofilm transmission between a broad range of donor and receiver surfaces. The instrument consists of a stainless steel pipe (donor surface) attached to a holder that be moved downward over the total length of receiver rubber tube. Thus, a biofilm can be grown at the luminal side of the pipe, and this device can use to investigate shear‐induced biofilm transmission to the extraluminal side of the receiver surface by quantifying the number of bacteria transmitted over the length of the silicone rubber tube (see schematic Fig. 1 in Gusnaniar *et al*. (2017)).

To evaluate the performance of the instrument, Gusnaniar *et al*. (2017) used two staphylococcal species, *Staphylococcus epidermidis* and *Staphylococcus aureus*, as model organisms. The authors observed that transmission occurred gradually over the length of receiver surface, concluding that such transmission was associated with the lack of biofilm cohesiveness rather than due to a failure of adhesiveness to the donor surface. Importantly, the device designed by Gusnaniar *et al*. (2017) achieved high reproducibility in the data obtained as compared with previous approaches described in the bibliography. Significantly, this technology can be used to evaluate how different bacterial genotypes (i.e. screening bacterial mutants), environmental cues and antibiofilm agents (i.e. bacteriophage cocktails) affect biofilm stability, dispersal and transmission (Donlan, 2002; Abee *et al*., 2011; Domenech *et al*., 2012; Alves *et al*., 2016; Petrova and Sauer, 2016; Rice *et al*., 2016; Guilhen *et al*., 2017). For example, Gusnaniar *et al*. (2017) investigated the role of extracellular polymeric substances (EPS) in the transmissibility of biofilms. The authors found that EPS‐containing biofilms showed a decreased friction coefficient and, as a consequence, biofilms were transmitted at higher levels over the length of the receiver surface when high shearing speed was applied.

Taken together, the technology presented by Gusnaniar *et al*. (2017) may facilitate the characterization of biofilm's properties throughout development, deciphering the role of different genetic and environmental factors on the dispersal and transmissibility of biofilms. The original prototype can be improved to finally develop a technology that allows the automated modification of additional shearing parameters (i.e. strength, friction angle) to more precisely investigate how these factors affect biofilm transmission over a wide range of surfaces and materials.

## Conflict of interest

None declared.
